# First-Principles Investigation on the Tunable Electronic Structures and Photocatalytic Properties of AlN/Sc_2_CF_2_ and GaN/Sc_2_CF_2_ Heterostructures

**DOI:** 10.3390/molecules29143303

**Published:** 2024-07-12

**Authors:** Meiping Liu, Yidan Lu, Jun Song, Benyuan Ma, Kangwen Qiu, Liuyang Bai, Yinling Wang, Yuanyuan Chen, Yong Tang

**Affiliations:** 1 School of Intelligent Manufacturing, Huanghuai University, Zhumadian 463000, China; 2 Henan Key Laboratory of Smart Lighting, School of Energy Engineering, Huanghuai University, Zhumadian 463000, China; 3 Polymer, Recycling, Industrial, Sustainability and Manufacturing (PRISM), Technological University of the Shannon: Midlands Midwest, N37 HD68 Athlone, Ireland

**Keywords:** AlN/Sc_2_CF_2_ and GaN/Sc_2_CF_2_ heterostructures, electronic structures, first-principles calculations, photocatalytic water splitting

## Abstract

Heterostructure catalysts are highly anticipated in the field of photocatalytic water splitting. AlN/Sc_2_CF_2_ and GaN/Sc_2_CF_2_ heterostructures are proposed in this work, and the electronic structures were revealed with the first-principles method to explore their photocatalytic properties for water splitting. The results found that the thermodynamically stable AlN/Sc_2_CF_2_ and GaN/Sc_2_CF_2_ heterostructures are indirect semiconductors with reduced band gaps of 1.75 eV and 1.84 eV, respectively. These two heterostructures have been confirmed to have type-Ⅰ band alignments, with both VBM and CBM contributed to by the Sc_2_CF_2_ layer. AlN/Sc_2_CF_2_ and GaN/Sc_2_CF_2_ heterostructures exhibit the potential for photocatalytic water splitting as their VBM and CBM stride over the redox potential of water. Gibbs free energy changes in HER occurring on AlN/Sc_2_CF_2_ and GaN/Sc_2_CF_2_ heterostructures are as low as −0.31 eV and −0.59 eV, respectively. The Gibbs free energy change in HER on the AlN (GaN) layer is much lower than that on the Sc_2_CF_2_ surface, owing to the stronger adsorption of H on AlN (GaN). The AlN/Sc_2_CF_2_ and GaN/Sc_2_CF_2_ heterostructures possess significant improvements in absorption range and intensity compared to monolayered AlN, GaN, and Sc_2_CF_2_. In addition, the band gaps, edge positions, and absorption properties of AlN/Sc_2_CF_2_ and GaN/Sc_2_CF_2_ heterostructures can be effectively tuned with strains. All the results indicate that AlN/Sc_2_CF_2_ and GaN/Sc_2_CF_2_ heterostructures are suitable catalysts for photocatalytic water splitting.

## 1. Introduction

The efficient use of solar energy has been widely regarded as one of the most prominent approaches to addressing the issues of energy depletion and environmental pollution. Photocatalytic water splitting into H_2_ and O_2_ provides a more practical scheme for the efficient utilization of solar energy [1]. Numerous semiconductors have been developed to serve photocatalytic water splitting [2,3,4,5,6,7]. However, there are some basic but quite strict requirements for semiconductor photocatalysts [8,9]. Firstly, the valence band maximum (VBM) of a photocatalyst should be lower than the reduction potential of O_2_/H_2_O (E[O_2_/H_2_O]); meanwhile, its conduction band minimum (CBM) should exceed the reduction potential of H^+^/H_2_ (E[H^+^/H_2_]). In addition, photocatalysts also require superior light absorption in the visible region and low carrier recombination, which can provide enough photo-generated carriers for the hydrogen evolution reaction (HER) and oxygen evolution reaction (OER). Over the last few decades, some mono-component photocatalysts, such as TiO_2_ [10], ZnO [11], and BiVO_4_ [12], have been found with the capabilities for water splitting. Yet, these photocatalysts suffer from the disadvantages of poor visible light absorption and serious carrier recombination, limiting the application process of photocatalytic water splitting [13,14]. Therefore, the primary challenge that needs to be addressed for the widespread implementation of photocatalytic water splitting is to develop novel high-performance catalysts with an appropriate band gap, superior visible light absorption behavior, and a low carrier recombination rate [15]. Heterostructure photocatalysts [5,16], composed of multi-components, generally possess these aforementioned advantages and are among the catalysts most likely to be implemented in the future.

The emerging two-dimensional (2D) materials of atomic thickness have been discovered with notable potential for photocatalytic water splitting owing to their high cost-effectiveness, massive specific surface areas, and abundant active sites [17]. Transition metal carbides (MXene) are presently one of the most popular 2D materials, with prospective applications in solar energy conversion, such as photocatalysis and photovoltaics. Sc_2_CF_2_ is a semi-conductive MXene, and it has been predicted to have an electron mobility and thermal conductivity of up to 10^3^ cm^2^*V*^−1^*s^−1^ and 472 W*m^−1^*K^−1^, respectively [18]. Even though its high carrier mobility and thermal conductivity are highly beneficial for photocatalytic water splitting, the VBM of Sc_2_CF_2_ exceeds E[O_2_/H_2_O], resulting in its inability to catalyze to the OER [19]. As other key members of 2D materials, AlN and GaN have recently been prepared by the MOCVD, PVT, MBE, and MEEG techniques [20,21,22,23]. These two nitrides are reported to exhibit unique optoelectronic and mechanical performance [24,25], and they have been considered as candidates for future optoelectronic devices [26,27,28,29,30,31,32]. For example, 2D AlN shows great promise in ultraviolet optoelectronic and laser diode applications [20,33], while 2D GaN has been investigated as a decent semiconductor for heterostructures and photocathodes [34,35]. Interestingly, the energy levels of VBM and CBM meet the requirements of photocatalytic water splitting [36]. However, they are all wide-band gap semiconductors with an ultraviolet absorption response only. Thus, their performances of photocatalytic water splitting are far below the requirement of real-world applications. Furthermore, several previous reports have proven the successes of heterostructures in optimizing the photocatalytic performance of MXene and nitrides [37,38,39]; nevertheless, the observations of AlN/Sc_2_CF_2_ and GaN/Sc_2_CF_2_ heterostructures are still missing. Considering the favorable electronic nature of previously synthesized Sc_2_CF_2_ and nitrides (AlN, GaN), it therefore makes sense to investigate the novel properties and prospective photocatalytic performance of AlN/Sc_2_CF_2_ and GaN/Sc_2_CF_2_ heterostructures.

In this work, AlN/Sc_2_CF_2_ and GaN/Sc_2_CF_2_ heterostructures were developed, and their electronic structures were investigated using the first-principles method to reveal the application potential for photocatalytic water splitting. Firstly, the stabilities of these two heterostructures were confirmed with binding energy (*E*_b_) calculations and ab initio molecular dynamic (AIMD) simulations. Then, the results demonstrated that AlN/Sc_2_CF_2_ and GaN/Sc_2_CF_2_ heterostructures possess type-Ⅰ band alignments. In addition, the charge density difference, band edge positions, free energy for HER, and absorption properties were also studied, indicating their promising abilities for photocatalytic water splitting. Finally, the effects of strain on the aforementioned performances were also revealed. The findings in this work extend the insights of novel photocatalysts and provide the theoretical foundation for experimental research to develop high-performance photocatalysts for water splitting.

## 2. Results and Discussions

**Figure 1:**
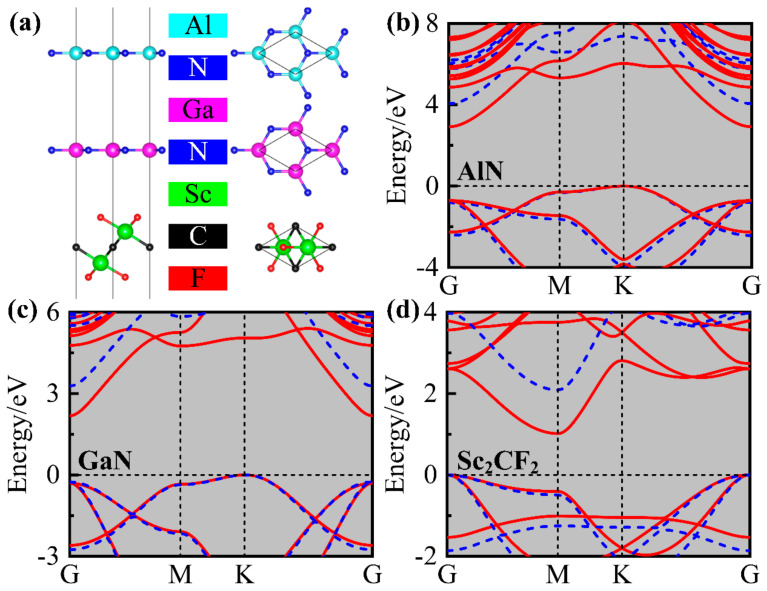
(**a**) The optimized AlN, GaN, and Sc_2_CF_2_ monolayers. The band structures for (**b**) AlN, (**c**) GaN, and (**d**) Sc_2_CF_2_ monolayers. The red-solid and blue-dotted lines in the band structures are the results using the PBE and HSE06 functional, respectively.

Firstly, the geometries and electronic structures of AlN, GaN, and Sc_2_CF_2_ monolayers were studied. The three optimized honeycomb geometries based on PBE functional are shown in Figure 1a, and the corresponding lattice constants were determined to be 3.22 Å, 3.29 Å, and 3.17 Å, respectively, matching up with earlier results [19,40,41,42]. The band structures of Sc_2_CF_2_, AlN, and GaN monolayers, calculated using the PBE and HSE06 functional, are exhibited in Figure 1b–d. It is clear from the band structures that all three monolayers are indirect band gap semiconductors. The VBM and CBM of the AlN (GaN) monolayer can observed at the K-point and G-point, respectively; however, the positions of VBM and CBM for the Sc_2_CF_2_ monolayer are generated at the M-point and K-point. The values of the band gap determined by the PBE functional are 2.92 eV, 2.19 eV, and 1.02 eV, whereas the in-turn results based on the HSE06 functional are 4.01 eV, 3.28 eV, and 2.09 eV. All the findings of the band gap are almost the same as the previous reports [36,38,43].

**Figure 2:**
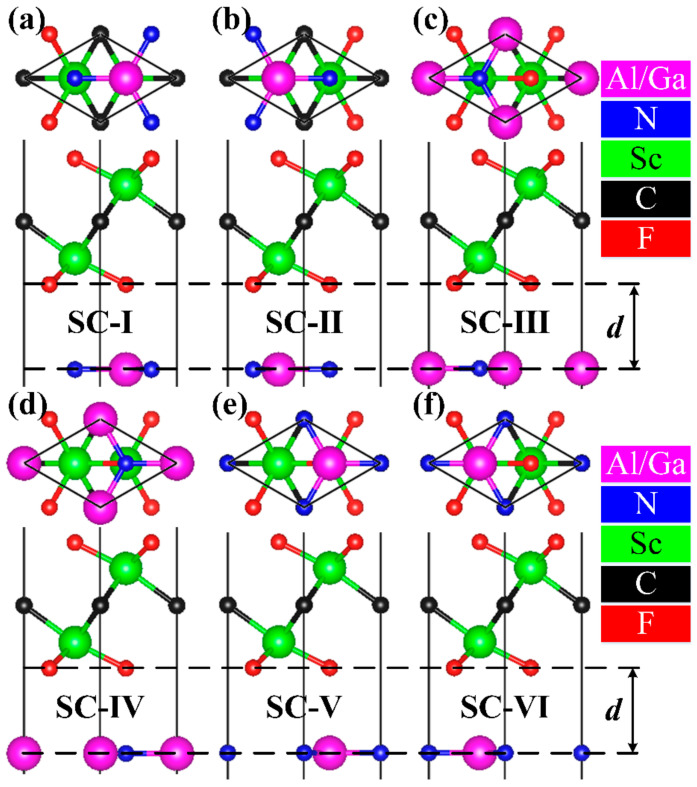
(**a**−**f**) The bottom and side views of AlN/Sc_2_CF_2_ (GaN/Sc_2_CF_2_) heterostructures. SC-I to SC-VI correspond to the six stacking configurations.

Since there are almost similar lattice constants between the AlN (GaN) layer and the Sc_2_CF_2_ layer, the AlN/Sc_2_CF_2_ (GaN/Sc_2_CF_2_) heterostructures are constructed by stacking and translating the AlN (GaN) layer on the Sc_2_CF_2_ layer. As presented in Figure 2, there are six generally observed stacking configurations (SCs) of AlN/Sc_2_CF_2_ and GaN/Sc_2_CF_2_ heterostructures that have been taken into account. Following sufficient structural relaxation based on the PBE functional, the lattice constants of AlN/Sc_2_CF_2_ and GaN/Sc_2_CF_2_ heterostructures given in Table 1 are similar to those of AlN and GaN monolayers, respectively, which may be attributed to the higher mechanical properties of AlN and GaN monolayers compared to that of the Sc_2_CF_2_ monolayer. The interlayer distances *d* for the AlN/Sc_2_CF_2_ and GaN/Sc_2_CF_2_ heterostructures within SC-I are the smallest, with the values of 2.71 Å and 2.82 Å, respectively. The minimum interlayer distance suggests the probable presence of significant interlayer coupling, which may profoundly influence the stabilities and electronic structures of the AlN/Sc_2_CF_2_ and GaN/Sc_2_CF_2_ heterostructures. To confirm the most feasible SC, the values of *E*_b_ for AlN/Sc_2_CF_2_ and GaN/Sc_2_CF_2_ heterostructures have been given as follows:

(1) $$E_{b}=\frac{E_{\mathrm{het}}-{\;E}_{\mathrm{AlN}}\;(E_{\mathrm{GaN}})-E_{\mathrm{Sc}2\mathrm{CF}2}}{S}$$

where *E*_het_, *E*_AlN_ (E_GaN_), and *E*_Sc2CF2_ represent the energy of the heterostructure, AlN (GaN) monolayer, and Sc_2_CF_2_ monolayer, respectively, whereas *S* is the interface area. All the values in Table 1 for the AlN/Sc_2_CF_2_ and GaN/Sc_2_CF_2_ heterostructures are negative, indicating their thermodynamic stabilities. As expected, SC-I was checked to be optimal, as the corresponding AlN/Sc_2_CF_2_ and GaN/Sc_2_CF_2_ heterostructures had the lowest values of −22.17 meV*Å^−2^ and −30.82 meV*Å^−2^. The results of *d* and *E*_b_ suggest that the AlN and GaN can establish stable heterostructures with Sc_2_CF_2_ through van der Waals bonding [44]. Therefore, subsequent research concentrates mainly on the AlN/Sc_2_CF_2_ and GaN/Sc_2_CF_2_ heterostructures in SC-I. 

**Figure 3:**
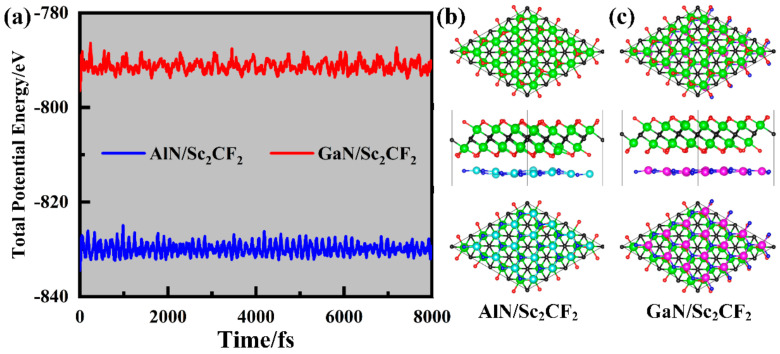
(**a**) The total energies of AlN/Sc_2_CF_2_ and GaN/Sc_2_CF_2_ heterostructures in the AIMD simulation at a temperature of 300K, as well as their (**b**) initial and (**c**) final snapshots.

Furthermore, the AIMD simulations were performed to ascertain the thermodynamic stabilities of AlN/Sc_2_CF_2_ and GaN/Sc_2_CF_2_ heterostructures in SC-I. Overall, the results of the total potential energy shown in Figure 3a demonstrate that the energy convergence of AlN/Sc_2_CF_2_ and GaN/Sc_2_CF_2_ heterostructures remains stable throughout the entire AIMD simulation process, with only minor fluctuations. At the same time, the final snapshots shown in Figure 3b,c still retain their complete structures without clear structural distortion, illustrating their thermodynamic stabilities. In addition, the phonon spectra of the AlN/Sc_2_CF_2_ and GaN/Sc_2_CF_2_ heterostructures in SC-I, shown in Figure S1, further confirm the dynamical stabilities.

**Figure 4:**
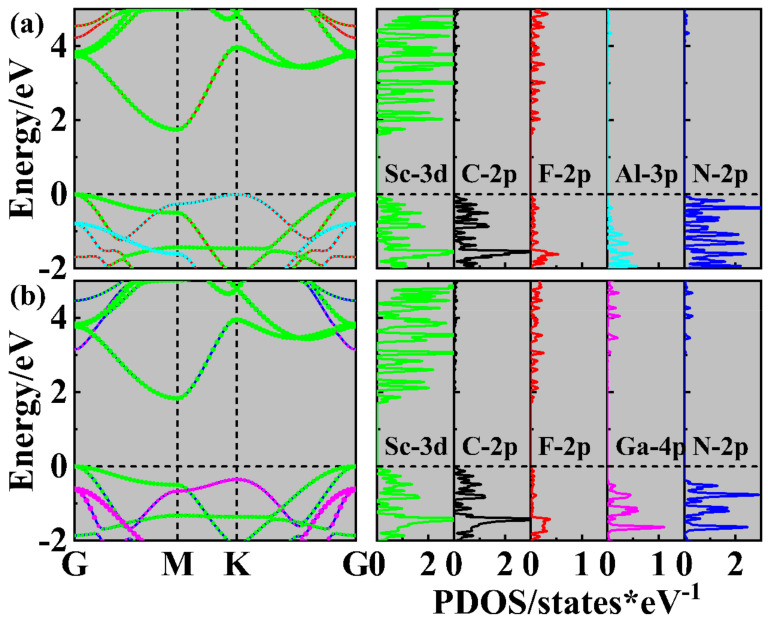
The projected band structure and PDOS of (**a**) AlN/Sc_2_CF_2_ and (**b**) GaN/Sc_2_CF_2_ heterostructures using the HSE06 functional. In the band structures, the green circles represent the contributions of the Sc_2_CF_2_ monolayer, while the cyan and magenta circles show those of the AlN and GaN layers, respectively.

Figure 4 shows the projected band structures and projected density of states (PDOSs) for AlN/Sc_2_CF_2_ and GaN/Sc_2_CF_2_ heterostructures. Both the AlN/Sc_2_CF_2_ and GaN/Sc_2_CF_2_ heterostructures are indirect semiconductors with band gap values of 1.75 eV and 1.84 eV, respectively. As indicated in the projected band structures, the VBM and CBM of AlN/Sc_2_CF_2_ and GaN/Sc_2_CF_2_ heterostructures are primarily populated by the Sc_2_CF_2_ layer, resulting in the formation of type-I band alignments in these heterostructures. For the two heterostructures, according to the PDOS, it is evident that Sc-3*d* orbitals make a substantial contribution to CBM and VBM, while the role of C-2*p* orbitals in the VBM is more significant than that in CBM, whereas the F-2*p* orbitals contribute an insignificant amount compared to both the VBM and CBM. However, the GaN layer provides a more substantial contribution to the VBM of the GaN/Sc_2_CF_2_ heterostructure compared to the AlN layer in the AlN/Sc_2_CF_2_ heterostructure. This difference may be due to the higher energy level of VBM in GaN compared to AlN. Furthermore, the band structures of the AlN/Sc_2_CF_2_ and GaN/Sc_2_CF_2_ heterostructures in all six SC are calculated by the PBE and HSE06 functional. The band structures in Figures S2–S5 indicate that all the heterostructures are indirect band gap semiconductors. As exhibited in Table 1, the results obtained from the PBE and HSE06 suggest that SC has no clear influence on the band gap.

**Figure 5:**
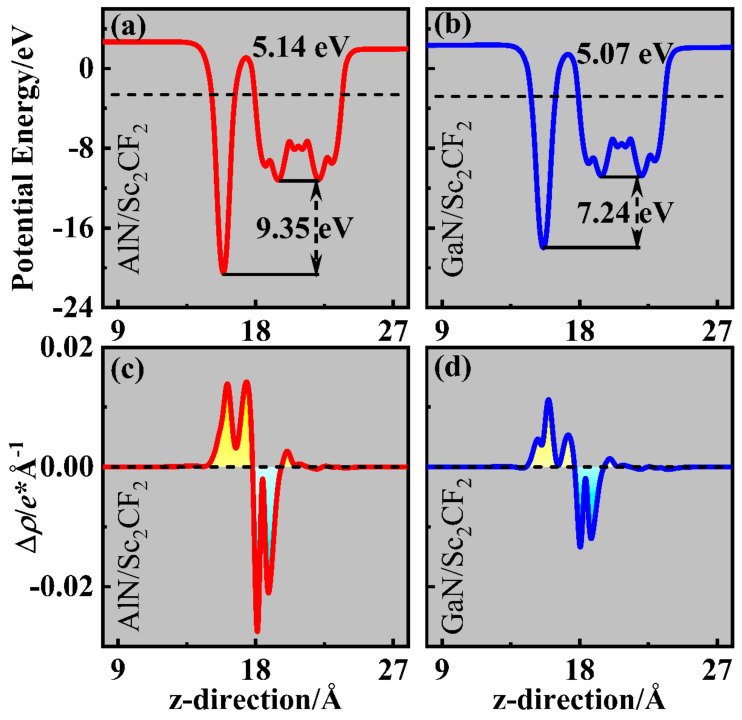
The potential and charge density difference in (**a**,**c**) AlN/Sc_2_CF_2_ and (**b**,**d**) GaN/Sc_2_CF_2_ heterostructures. The isosurface of the insert was set to 3 × 10^−4^ e*Å^−3^.

The effect of interlayer coupling is particularly considerable on the electronic structure of heterostructures [45]. While the AlN (GaN) monolayer comes into contact with the Sc_2_CF_2_ monolayer, electrons spontaneously migrate from the layer with a smaller work function (*W*) to another layer until their Fermi levels approach equally. The values of *W* for AlN, GaN, and Sc_2_CF_2_ monolayers can be calculated with expression (2) to comprehend the electron migration mechanism:

(2) $$W=E_{\mathrm{vac}}-E_{F}$$

where *E*_vac_ and *E*_F_ refer to the energies of the vacuum level and the Fermi level, respectively. As shown in Figure S6, the corresponding values of W for the AlN, GaN, and Sc_2_CF_2_ monolayers are 5.23 eV, 5.18 eV, and 4.95 eV. These findings indicate the electron transfer from the Sc_2_CF_2_ monolayer to the AlN and GaN layers. Thus, the electron and hole concentration regions emerge near the AlN (GaN) and Sc_2_CF_2_ layers, respectively. In Figure S7, because of the electrostatic induction [46,47,48], electrons in the AlN (GaN) layer are repelled by the electrons on the surface of Sc_2_CF_2_, causing upward band bending of the AlN (GaN) layer, while the band of Sc_2_CF_2_ bends downward. When the heterostructures are exposed to light irradiation, electrons excited on the CBM of the AlN (GaN) layer can spontaneously flow to the CBM of the Sc_2_CF_2_ layer; then, these electrons can be used for the HER. Owing to the potential barriers [37], holes are retained in the VBM of AlN (GaN) and Sc_2_CF_2_ layers for OER. Bader charge calculations revealed that 0.095 |*e*| electrons flow from the Sc_2_CF_2_ monolayer to the AlN monolayer, while the number of electrons transferring to the GaN monolayer was 0.041|*e*| [49]. As illustrated in Figure 5a,b, AlN/Sc_2_CF_2_ and GaN/Sc_2_CF_2_ heterostructures were found with the *W* of 5.14 eV and 5.07 eV, respectively. Moreover, the potential drops introduced by the electron migration were observed in AlN/Sc_2_CF_2_ and GaN/Sc_2_CF_2_ heterostructures, with values of 9.35 eV and 7.24 eV, respectively. The potential drop establishes a built-in electric field orientating from the AlN (GaN) layer and Sc_2_CF_2_ layer. The potential drops in AlN/Sc_2_CF_2_ and GaN/Sc_2_CF_2_ heterostructures are much greater than those of the past reported InSe/MoS_2_ (2.55 eV) [50], GaN/GeS (less than 0.5 eV) [51], and CrSSe/MoS_2_ (2.30 eV) [52] heterostructures. The large potential drops result in the generation of strong built-in electric fields in AlN/Sc_2_CF_2_ and GaN/Sc_2_CF_2_ heterostructures, and the built-in electric fields are beneficial for carrier separation in photocatalysis and photovoltaic processes [53]. In practical applications, defect and doping engineering can be implemented in the interfaces of AlN/Sc_2_CF_2_ and GaN/Sc_2_CF_2_ heterostructures to enhance the strength of the built-in electric field, which helps promote the separation of charge carriers. In addition, the charge density difference *ρ* can be used to track the charge transfer and obtain information on the interactions between different components in heterostructures [54]. The *ρ* for AlN/Sc_2_CF_2_ and GaN/Sc_2_CF_2_ heterostructures are calculated by expression (3):

(3) $$∆\rho =\rho_{\mathrm{het}}\;-\;\rho_{\mathrm{AlN}}(\rho_{\mathrm{GaN}})\;\;-\;\rho_{\mathrm{Sc}2\mathrm{CF}2}$$

where *ρ*_het_, *ρ*_AlN_ (*ρ*_GaN_) and *ρ*_Sc2CF2_ express the charge densities of the AlN/Sc_2_CF_2_ (GaN/Sc_2_CF_2_) heterostructure and AlN (GaN) and Sc_2_CF_2_ monolayers, respectively. From the results plotted in Figure 5c,d, the electron migration from the Sc_2_CF_2_ layer to the AlN (GaN) layer is evidently noticeable, and the transfer behavior is more pronounced in the AlN/Sc_2_CF_2_ heterostructure, conforming to the previous statements.

**Figure 6:**
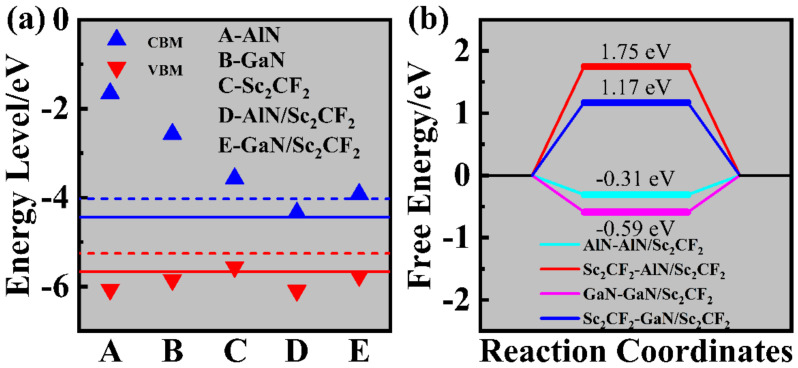
(**a**) The energy levels of VBM and CBM for the free monolayers and heterostructures. The black lines and dashed lines represent the redox potentials for water splitting at pH = 0 and 7, respectively. (**b**) Gibbs free energy diagram for HER on the AlN/Sc_2_CF_2_ and GaN/Sc_2_CF_2_ heterostructures, respectively.

To reveal the potential of AlN/Sc_2_CF_2_ and GaN/Sc_2_CF_2_ heterostructures for photocatalytic water splitting, the energy levels of VBM and CBM for two heterostructures and three freestanding monolayers were evaluated with the method proposed by Toroker [55]. As can be seen from Figure 6a, the positions of VBM and CBM for the AlN and GaN monolayers match the demands of photocatalytic water splitting, and their wide band gaps allow them to work within the extensive pH range. For the Sc_2_CF_2_ monolayer, its CBM and VBM are beyond E[H^+^/H_2_] and E[O_2_/H_2_O], respectively, indicating that it has the catalytic ability for HER but is incapable of OER. The CBM and VBM of the AlN/Sc_2_CF_2_ heterostructure are located at −4.29 eV and −6.04 eV, respectively, whereas the GaN/Sc_2_CF_2_ heterostructure possesses the values of −3.92 eV and −5.76 eV. Moreover, the GaN/Sc_2_CF_2_ heterostructure has been identified with a wide work pH range from 0 to 7. Figure S8 shows the band alignments of AlN/Sc_2_CF_2_ and GaN/Sc_2_CF_2_ heterostructures, and it is evident that the AlN, GaN, and Sc_2_CF_2_ monolayers in heterostructures are assessable for photocatalytic water splitting. The Gibbs free energy change Δ*G* of HER is valuable to estimate the photocatalytic property from a thermodynamic perspective [56], and the Δ*G* for HER occurring on both AlN, GaN, and Sc_2_CF_2_ surfaces in the heterostructure is calculated to assess the feasibility of HER, driven by the AlN/Sc_2_CF_2_ and GaN/Sc_2_CF_2_ heterostructures. Nine possible sites for H-atom adsorbing on the heterostructure, as shown in Figure S9, were considered in this work. The calculation details and stable adsorption structures (Figure S10) are provided in the Supplementary Materials. The structure that had the lowest energy was chosen to be the most stable adsorption site. The H-atom was adsorbed on the F atom of the Sc_2_CF_2_ layer, similar to the surfaces of AlN and GaN, it was located above the N atom. The results in Figure 6b demonstrate that the Δ*G* for HER on the Sc_2_CF_2_ layer in AlN/Sc_2_CF_2_ and GaN/Sc_2_CF_2_ heterostructures was 1.75 eV and 1.17 eV. As these present values of Δ*G* are smaller than the 2.63 eV for N-Ni_3_S_2_/NF [57], which is available for driving HER experimentally, HER on the Sc_2_CF_2_ surface in the AlN/Sc_2_CF_2_ and GaN/Sc_2_CF_2_ heterostructures should likewise be experimentally practicable. However, the HER occurring on the AlN and GaN surfaces in AlN/Sc_2_CF_2_ and GaN/Sc_2_CF_2_ heterostructures is more favorable, for which the values of Δ*G* are −0.31 eV and −0.59 eV, respectively. The Δ*G* values of HER on AlN and GaN surfaces are close to those of cobalt phosphide catalysts reported with good performances of photocatalytic water splitting [58] and smaller than that of the freestanding AlN monolayer [59]. The smaller Δ*G* for HER on the AlN and GaN surfaces in heterostructures may be attributed to their large overpotentials, as shown in Table S2 [60]. As is known from Figure S11, the *p*-band center was calculated to further comprehend better HER performance on AlN and GaN surfaces. The values of the N-2*p* band center for H adsorption on AlN and GaN surfaces are −1.77 and −3.02 eV, while the center values of the F-2*p* band were predicted to be −6.98 and −6.53 eV for adsorption on Sc_2_CF_2_ in AlN/Sc_2_CF_2_ and GaN/Sc_2_CF_2_ heterostructures. A lower band center means the stronger adsorption strength of H on the N atom compared to that on the F atom [61]. Therefore, the AlN/Sc_2_CF_2_ and GaN/Sc_2_CF_2_ heterostructures with appropriate Δ*G* exhibit attractive application potential as photocatalysts for water splitting to produce clean hydrogen energy.

Strain is a common effect at the interface of the heterostructure that considerably impacts its electronic structure [62,63]. The band structures and absorption coefficients of strained AlN/Sc_2_CF_2_ and GaN/Sc_2_CF_2_ heterostructures are calculated using HSE06 functional to explore the effect of biaxial strain on their electronic structures and absorption properties. The strain *ε* is defined as follows:

(4) $$\varepsilon =\frac{a\;-a_{0}}{a_{0}}$$

where *a* and *a*_0_ stand for the lattice constants of strained and free heterostructures. Six strains of −6%, −4%, −2%, 2%, 4%, and 6% were applied to the heterostructures. The band structures and PDOS of strained AlN/Sc_2_CF_2_ and GaN/Sc_2_CF_2_ heterostructures are exhibited in Figures S12–S15.

**Figure 7:**
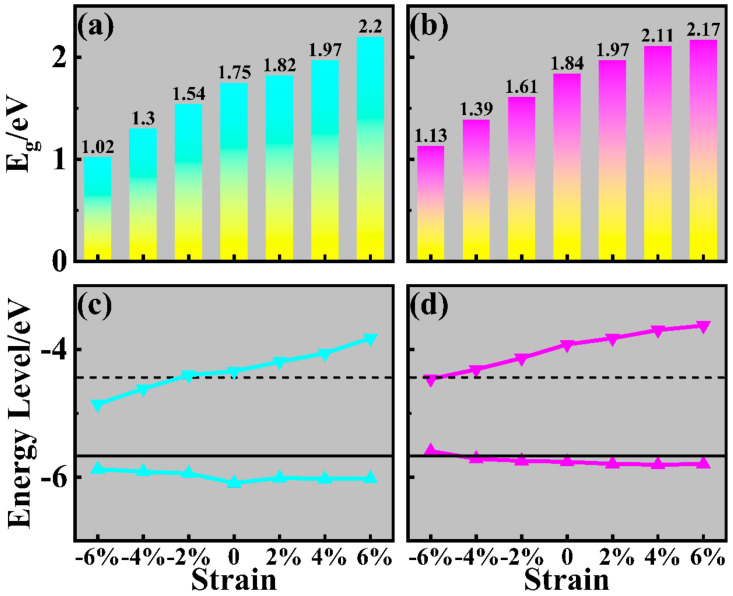
The band gaps and band edge positions of strained (**a**,**c**) AlN/Sc_2_CF_2_ and (**b**,**d**) GaN/Sc_2_CF_2_ heterostructures.

It is evident that all heterostructures maintain an indirect band gap feature with type-Ⅰ band alignment since the VBM and CBM of strained heterostructures are contributed to by AlN and GaN, respectively. As shown in Figure 7a,b, the effect of strains on the band gaps of AlN/Sc_2_CF_2_ and GaN/Sc_2_CF_2_ heterostructures is clear, with lattice compression and tension reducing and increasing the bandgap, respectively. With strains varying from −6% to 6%, the values of band gap for the strained AlN/Sc_2_CF_2_ heterostructures increase from 1.02 eV to 2.2 eV, whereas those of the strained GaN/Sc_2_CF_2_ heterostructures change from 1.13 eV to 2.17 eV. The energy levels of VBM and CBM for the strained AlN/Sc_2_CF_2_ and GaN/Sc_2_CF_2_ heterostructures are displayed in Figure 7c,d. The tendency of band gaps for strained heterostructures can be understood from the opposite effects of the lattice strain on the energy levels of VBM and CBM. What the opposite effects mean is that the lattice tension causes the VBM energy level to decrease, and it also leads to an increase in the CBM energy level. Hence, the energy differences between VBM and CBM expand, leading to an increase in band gaps. According to the band edge positions, the −2%-strained AlN/Sc_2_CF_2_ heterostructure and the −2%-strained GaN/Sc_2_CF_2_ heterostructure still maintain their capabilities for photocatalytic water splitting. As the lattice constants of AlN/Sc_2_CF_2_ and GaN/Sc_2_CF_2_ heterostructures expand, their overpotentials increase, which potentially leads to improved photocatalytic activities.

**Figure 8:**
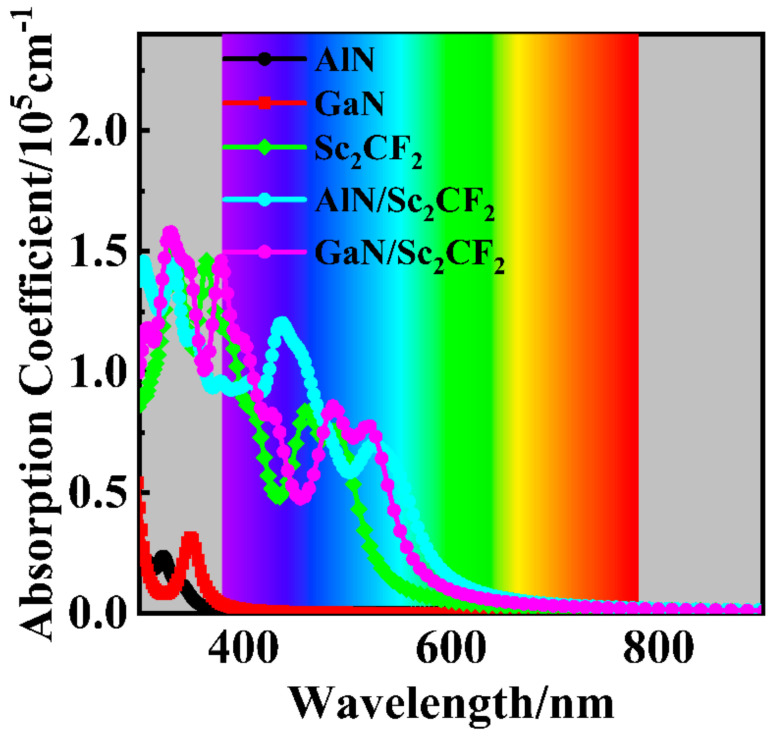
Absorption coefficients of AlN/Sc_2_CF_2_ and GaN/Sc_2_CF_2_ heterostructures, as well as those of AlN, GaN, and Sc_2_CF_2_ monolayers.

Light absorption performance is an important indicator for photocatalysts, which determines the upper limit of photogenerated carriers used for subsequent HER and OER. The absorption coefficients of AlN/Sc_2_CF_2_ and GaN/Sc_2_CF_2_ heterostructures and their freestanding monolayer components can be calculated using expression (5):

(5) $$\alpha \left(\omega \right)=\sqrt{2}\omega \sqrt{\sqrt{\varepsilon_{1}^{2}\left(\omega \right)+\varepsilon_{2}^{2}\left(\omega \right)}-\varepsilon_{1}\left(\omega \right)}$$

where ω is the photon frequency, and *ε*_1_(ω) and *ε*_2_(ω) stand for the real and imaginary parts of the dielectric function. As proven by Figure 8, the absorption behaviors of AlN and GaN monolayers are only observed in the ultraviolet region with low intensities due to their wide band gaps. The absorption activity of the Sc_2_CF_2_ monolayer is clear, with strong absorption intensity in the visible region. As for AlN/Sc_2_CF_2_ and GaN/Sc_2_CF_2_ heterostructures, their absorption range expands due to the reduced band gaps. Since the Sc_2_CF_2_ layers occupy VBM and CBM in the heterostructures, the absorption behaviors of AlN/Sc_2_CF_2_ and GaN/Sc_2_CF_2_ heterostructures are comparable to that of the Sc_2_CF_2_ monolayer. However, the absorption intensities of heterostructures are enhanced, which might be ascribed to interlayer coupling [64,65]. It can be inferred that the superior absorption activities of AlN/Sc_2_CF_2_ and GaN/Sc_2_CF_2_ heterostructures will produce more carriers for HER and OER. Hence, these two heterostructures possess better performances in photocatalytic water splitting than those of AlN, GaN, and Sc_2_CF_2_ monolayers.

**Figure 9:**
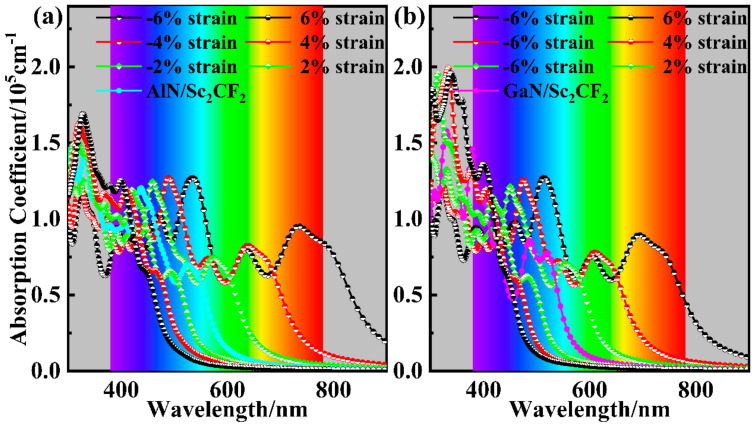
Absorption coefficients of the strained (**a**) AlN/Sc_2_CF_2_ and (**b**) GaN/Sc_2_CF_2_ heterostructures compared with the two freestanding heterostructures.

For strained AlN/Sc_2_CF_2_ and GaN/Sc_2_CF_2_ heterostructures, the absorption coefficients shown in Figure 9 demonstrate the modulation of strains on their optical absorption performances. As previously stated, compressive strains decrease the band gaps of the heterostructures, resulting in the expanded ranges of absorption for the compressed AlN/Sc_2_CF_2_ and GaN/Sc_2_CF_2_ heterostructures. The absorption ranges of tensile-strained AlN/Sc_2_CF_2_ and GaN/Sc_2_CF_2_ heterostructures become narrower as their band gaps increase. However, there is another noticeable fact in that the absorption intensities of tensile-strained AlN/Sc_2_CF_2_ and GaN/Sc_2_CF_2_ heterostructures, particularly in the UV range, are superior to those of compressed systems. The enhanced absorption intensity can be attributed to the changes in the charge density difference in the AlN/Sc_2_CF_2_ and GaN/Sc_2_CF_2_ heterostructures under different strains, as shown in Figure S16. It is evident that the changes in lattice have a significant impact on interlayer coupling. Specifically, more charge transfer from the Sc_2_CF_2_ layer to the AlN (GaN) layer in the lattice expanded heterostructures, and the transfer behaviors weaken in the compressed AlN/Sc_2_CF_2_ and GaN/Sc_2_CF_2_ heterostructures. The changes in charge transfer may directly influence the strength of the built-in electric field and potential drop, which are responsible for facilitating the migration of photogenerated carriers, thereby enhancing the absorption intensity [45,66,67]. These findings have validated the enhanced absorption capabilities of AlN/Sc_2_CF_2_ and GaN/Sc_2_CF_2_ heterostructures, as well as the strained ones, indicating their considerable potential for applications in photocatalytic water splitting.

## 3. Computational Methods

All computational investigations in the present work were carried out using the Vienna ab initio simulation package (VASP) [68], which is based on the Density Functional Theory (DFT). The exchange–correlation functional was treated by the generalized gradient approximation within the Perdew–Burke–Ernzerhof (GGA-PBE) scheme [69]. Projector-augmented wave pseudopotentials (PAW) were utilized [70,71] with a cutoff energy of 500 eV. The lattice constant and atom position were fully relaxed based on the PBE functional until the energy and force fell to less than 10^−5^ eV and 0.01 eV*Å^−1^, respectively. The calculations of band structure and absorption coefficient were conducted with the Heyd–Scueria–Ernzerhof hybrid functional (HSE06) [72]. The van der Waals force, described with the DFT-D3 method [73], was considered in all calculations. The vacuum space of all 2D systems was set to 30 Å in the z-direction to shield neighboring interactions. The Gamma-centered scheme [74] k-points with grids of 15 × 15 × 1 and 21 × 21 × 1 were implemented to sample the Brillouin zone for structure optimization and property calculations. The NVT-ensembled AIMD simulations [75], using the algorithm of the Nosé–Hoover thermostat, were performed on the 4 × 4 × 1 supercell to confirm the thermal stabilities of AlN/Sc_2_CF_2_ and GaN/Sc_2_CF_2_ heterostructures. The simulation temperature of 300 K was set, and the total simulation duration was 6 ps with a 1 fs step time. The VASPKIT package [76] and VESTA code [77] were employed for pre- and post-visualization.

## 4. Conclusions

AlN/Sc_2_CF_2_ and GaN/Sc_2_CF_2_ heterostructures are proposed in this work, and their electronic structures were investigated using the first-principles method to explore their photocatalytic properties. These thermodynamic stable heterostructures show type-I band alignments and their corresponding band gaps were found to be 1.84 eV and 1.75 eV. Furthermore, the clear potential drops of 9.35 eV and 7.24 eV were found to be present in AlN/Sc_2_CF_2_ and GaN/Sc_2_CF_2_ heterostructures, which can generate built-in electric fields to promote carrier separation. The band edge positions of AlN/Sc_2_CF_2_ and GaN/Sc_2_CF_2_ heterostructures are suitable for photocatalytic water splitting. The Gibbs free energy change in HER that occurred on the AlN and GaN surfaces in two heterostructures was as low as −0.31 eV and −0.59 eV, respectively. The lower Gibbs free energy changes may be attributed to the stronger adsorption behaviors on AlN and GaN compared to that on Sc_2_CF_2_ in heterostructures. As for absorption performance, both the AlN/Sc_2_CF_2_ and GaN/Sc_2_CF_2_ heterostructures possess significant improvements in absorption range and intensity compared to the monolayered AlN, GaN, and Sc_2_CF_2_. Furthermore, strains can effectively tune the band gaps, edge positions, and absorption properties of AlN/Sc_2_CF_2_ and GaN/Sc_2_CF_2_ heterostructures. The capabilities of photocatalytic water splitting for AlN/Sc_2_CF_2_ and GaN/Sc_2_CF_2_ heterostructures are kept over a wide strain range. All the findings above suggest that the AlN/Sc_2_CF_2_ and GaN/Sc_2_CF_2_ heterostructures are promising catalyst candidates for photocatalytic water splitting. Some properties of carriers, such as their mobility, lifetime, and diffusion length, which play important roles in photocatalytic reactions, are still unknown and can be studied further in the future.

## Figures and Tables

**Table 1:** Results of lattice constant *a* (Å), interlayer distance *d* (Å), binding energy *E*_b_ (meV*Å^−2^), and band gap *E*_g_ (eV) for AlN/Sc_2_CF_2_ and GaN/Sc_2_CF_2_ heterostructures.

Heterostructure	Item		SC-I	SC-II	SC-III	SC-IV	SC-V	SC-VI
AlN/Sc_2_CF_2_	*a*		3.21	3.20	3.20	3.20	3.20	3.20
	*d*		2.71	3.38	2.93	3.37	2.84	3.03
	*E*_b_		−22.17	−8.74	−13.62	−9.69	−18.25	−10.54
	*E*_g_	PBE	0.79	0.85	0.86	0.86	0.85	0.85
		HSE06	1.75	1.70	1.71	1.70	1.70	1.70
GaN/Sc_2_CF_2_	*a*		3.25	3.22	3.24	3.22	3.22	3.21
	*d*		2.82	3.37	2.98	3.31	2.85	3.18
	*E*_b_		−30.82	−15.21	−24.24	−15.87	−27.19	−21.27
	*E*_g_	PBE	0.90	0.95	0.96	0.95	0.93	0.95
		HSE06	1.84	1.81	1.82	1.81	1.82	1.81

## Data Availability

The data presented in this study are available upon request from the corresponding author.

## Supplementary Materials

The following supporting information can be downloaded at https://www.mdpi.com/article/10.3390/molecules29143303/s1, Table S1. The valence band offset (VBO) and conduct band offset (CBO) of AlN/Sc_2_CF_2_ and GaN/Sc_2_CF_2_ heterostructures; Table S2. The overpotential values for HER of different surfaces in AlN/Sc_2_CF_2_ and GaN/Sc_2_CF_2_ heterostructures; Figure S1. The phonon spectrum of (a) AlN/Sc_2_CF_2_ and (b) GaN/Sc_2_CF_2_ heterostructures; Figure S2. The band structures of AlN/Sc_2_CF_2_ heterostructures by using the PBE functional; Figure S3. The band structures of AlN/Sc_2_CF_2_ heterostructures by using the HSE06 functional; Figure S4. The band structures of GaN/Sc_2_CF_2_ heterostructures by using the PBE functional; Figure S5. The band structures of GaN/Sc_2_CF_2_ heterostructures by using the HSE06 functional; Figure S6. The potentials for AlN, GaN, and Sc_2_CF_2_ monolayers; Figure S7. The carrier transfer mechanical of AlN/Sc_2_CF_2_ and GaN/Sc_2_CF_2_ heterostructures. The MN represents AlN and GaN; Figure S8. The band alignments of AlN/Sc_2_CF_2_ and GaN/Sc_2_CF_2_ heterostructures; Figure S9. All the possible adsorption sites of H-atom on the (a) AlN layer and (b) Sc_2_CF_2_ layer in AlN/Sc_2_CF_2_ heterostructure. The same adsorption sites also have been considered in GaN/Sc_2_CF_2_ heterostructure; Figure S10. The stable adsorption of H atom on the AlN (GaN) and Sc_2_CF_2_ surface in AlN/Sc_2_CF_2_ (GaN/Sc_2_CF_2_) heterostructure; Figure S11. The PDOS distribution of H adsorbed on the surfaces of AlN (GaN) and Sc_2_CF_2_ in AlN/Sc_2_CF_2_ (GaN/Sc_2_CF_2_) heterostructure, respectively; Figure S12. The band structures of strained AlN/Sc_2_CF_2_ heterostructures by using the HSE06 functional; Figure S13. The PDOS of strained AlN/Sc_2_CF_2_ heterostructures by using the HSE06 functional; Figure S14. The band structures of strained GaN/Sc_2_CF_2_ heterostructures by using the HSE06 functional; Figure S15. The PDOS of strained GaN/Sc_2_CF_2_ heterostructures by using the HSE06 functional; Figure S16. The charge density difference of the strained AlN/Sc_2_CF_2_ and GaN/Sc_2_CF_2_ heterostructures. References [78,79,80,81,82,83] are cited in Supplementary Materials.
