# Supplementary material for: First-Principles Investigation on the Tunable Electronic Structures and Photocatalytic Properties of AlN/Sc2CF2 and GaN/Sc2CF2 Heterostructures
Source: Molecules. 2024 Jul 12;29(14):3303. doi: 10.3390/molecules29143303 (PMC11279752; doi:10.3390/molecules29143303)
Supplement: Supplementary file 1 [file molecules-29-03303-s001.zip › molecules-3087476-supplementary.pdf]

# First-principles investigation on the tunable optoelectronic and photocatalytic properties of $MN/Sc_2CF_2$ (M = Al, Ga) heterostructures

Meiping Liu[1], Yidan Lu[2], Jun Song[2], Benyuan Ma[2], Kangwen Qiu[2], Liuyang Bai[2], Yinling Wang[2], Yuanyuan Chen[3*], Yong Tang[2,3*].

1 School of Intelligent Manufacturing, Huanghuai University, Zhumadian 463000, Henan, China;

2 Henan Key Laboratory of Smart Lighting, and School of Energy Engineering, Huanghuai University, Zhumadian 463000, Henan, China;

3 Polymer, Recycling, Industrial, Sustainability and Manufacturing (PRISM), Technological University of the Shannon: Midlands Midwest, Athlone, Westmeath N37 HD68, Ireland.

*Correspondence: yuanyuan.chen@tus.ie (Y.C); yong.tang@tus.ie (Y.T).

**Details of phonon spectra of heterostructures.**

The phonon spectra of $AlN/Sc_2CF_2$ and $GaN/Sc_2CF_2$ heterostructures were calculated with DFPT method[1,2], as implemented in PHONOPY code[3]. Due to the limitations of computation resource, the 4×4×1 supercells, which has 112 atoms, of $AlN/Sc_2CF_2$ and $GaN/Sc_2CF_2$ heterostructures were used for the calculations, and the KPOINTS was set to 5×5×1. The lattice constant and atom position were fully relaxed until the energy and force fell less than $10^{-8}$ eV and 0.001 eV*$Å^{-1}$, respectively.

**Details of Gibbs free energy calculation.**

The Gibbs free energy ($\Delta G$) of HER was calculated by the formula[4]:

$$\Delta G = \Delta E + \Delta E_{\mathrm{zpe}} - T\Delta S - \Delta G_{pH} - eU.$$

Here, $\Delta E$ is the energy difference of the adsorption reaction, $\Delta E_{\mathrm{zpe}}$ represents the energy difference of zero-point energy, and $\Delta S$ means the difference in entropy between the adsorbed state and the gas phase. The temperature $T$ in this work was set to 298.15K. The effect of pH on $\Delta G$ was not considered in this work, hence the default value of $\Delta G_{\mathrm{pH}}$ equals 0. $U$ refers to the applied potential.

The procedure of HER is described as follows[5]:

$$* + H^+ + e^- \rightarrow H*$$

in the above reaction steps, * represents the adsorbed $Sc_2CF_2/Ti_2CO_2$ heterostructure. O*, OH*, OOH*, and H* are the reaction intermediates.

**Details of the *p*-band center calculation.**

Similar to the *d*-band center, the p-band center of H adsorption on the F and N atoms in $AlN/Sc_2CF_2$ and $GaN/Sc_2CF_2$ heterostructures is defined as[6]:

$$\varepsilon_p = \frac{\int_{-\infty}^{0} \mathrm{E D}(E)\mathrm{d}E}{\int_{-\infty}^{0} \mathrm{D}(E)\mathrm{d}E}.$$

In the above expression, D($E$) is the DOS value of the F-2$p$ orbital corresponding to the energy $E$.

**Table S1.** The valence band offset (VBO) and conduct band offset (CBO) of $AlN/Sc_2CF_2$ and $GaN/Sc_2CF_2$ heterostructures.

| items | $AlN/Sc_2CF_2$ | $GaN/Sc_2CF_2$ |
|---|---|---|
| VBO | 0.09 | 0.35 |
| CBO | 2.43 | 1.31 |

**Table S2.** The overpotential values for HER of different surfaces in $AlN/Sc_2CF_2$ and $GaN/Sc_2CF_2$ heterostructures.

| Surface | AlN in $AlN/Sc_2CF_2$ | $Sc_2CF_2$ in $AlN/Sc_2CF_2$ | GaN in $GaN/Sc_2CF_2$ | $Sc_2CF_2$ in $GaN/Sc_2CF_2$ |
|---|---|---|---|---|
| Overpotential | 2.58 | 0.15 | 1.83 | 0.52 |

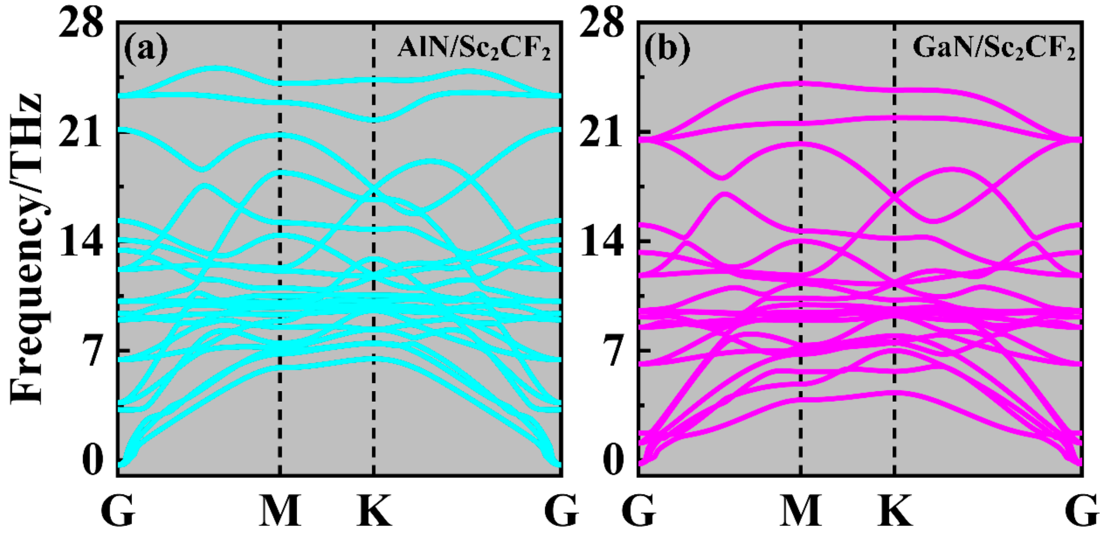


**Figure S1.** The phonon spectrum of (a) $AlN/Sc_2CF_2$ and (b) $GaN/Sc_2CF_2$ heterostructures.

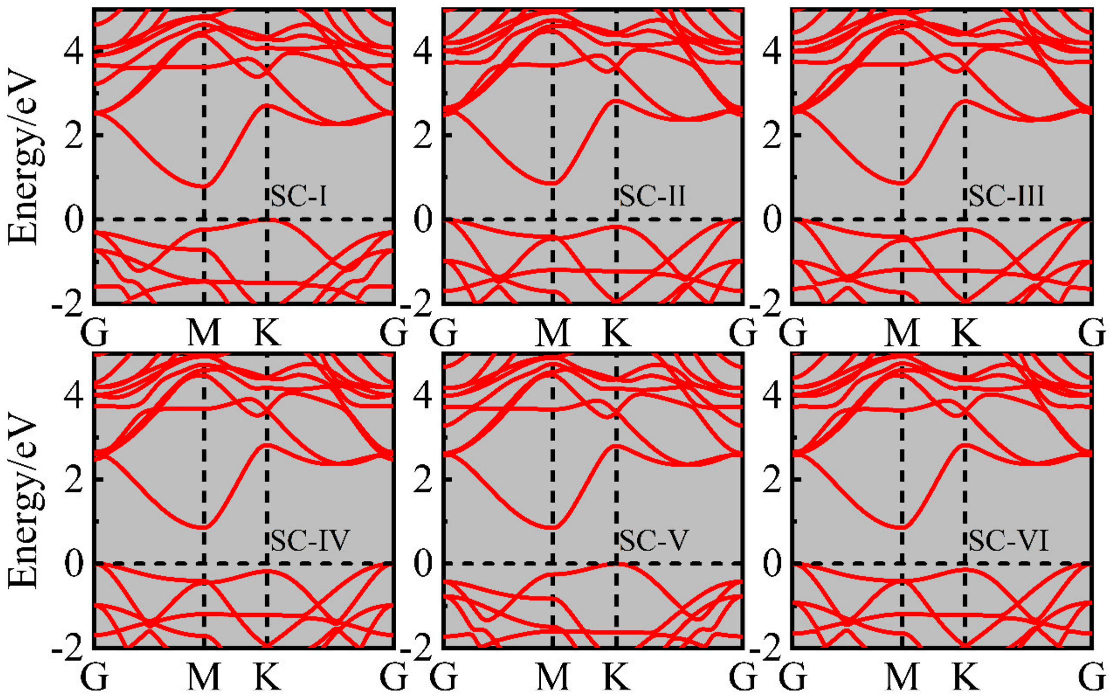


**Figure S2.** The band structures of $AlN/Sc_2CF_2$ heterostructures by using the PBE functional.

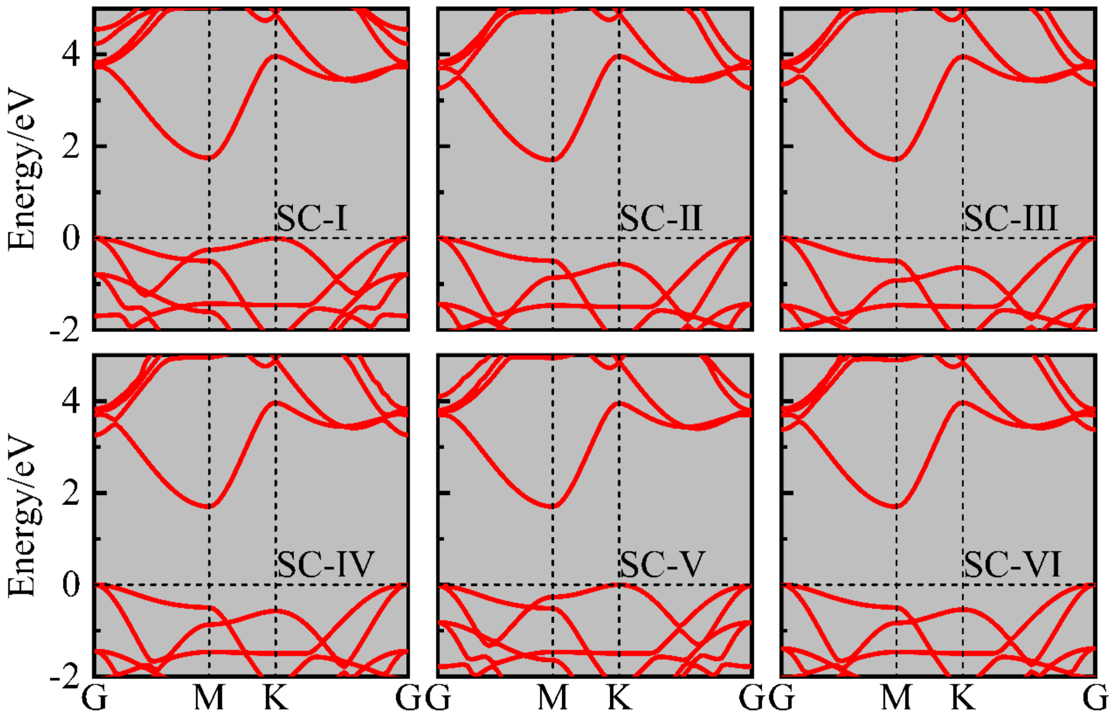


**Figure S3.** The band structures of AlN/$Sc_2CF_2$ heterostructures by using the HSE06 functional.

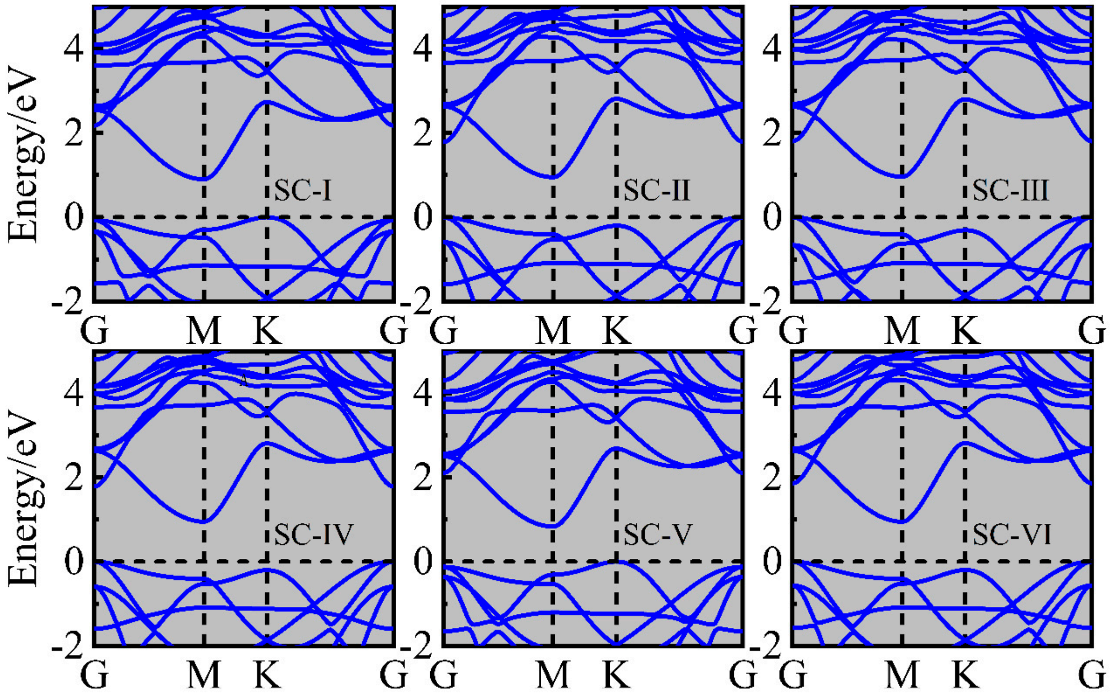


**Figure S4.** The band structures of GaN/$Sc_2CF_2$ heterostructures by using the PBE functional.

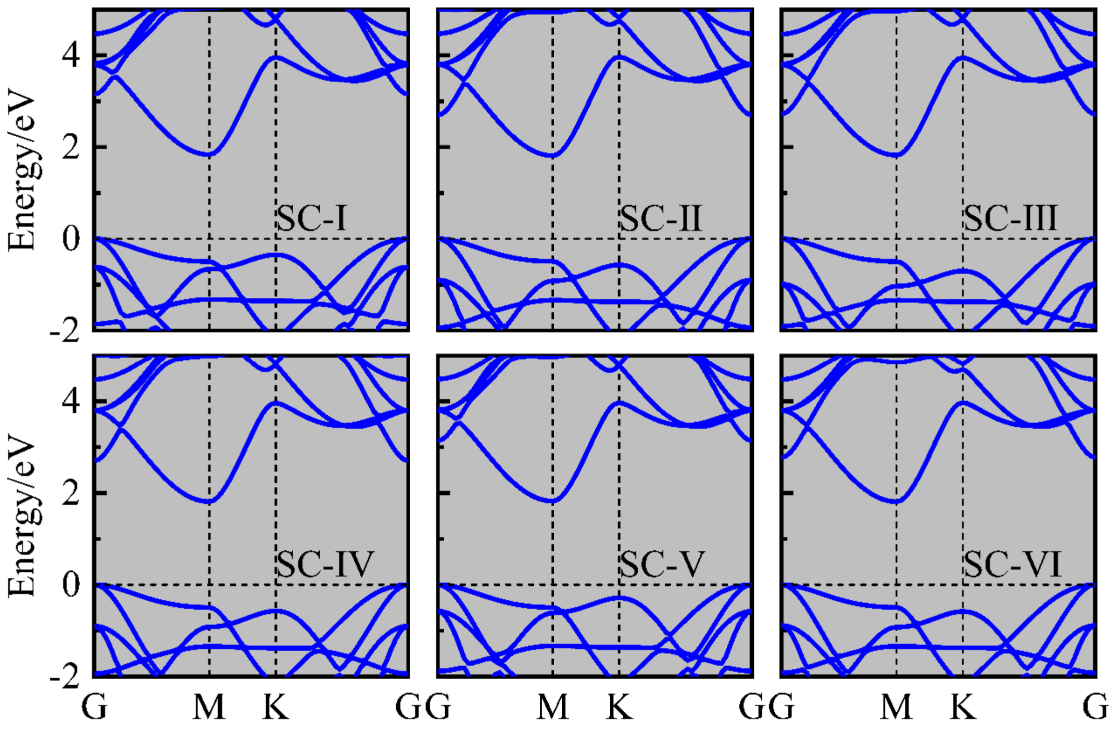


**Figure S5.** The band structures of GaN/$Sc_2CF_2$ heterostructures by using the HSE06 functional.

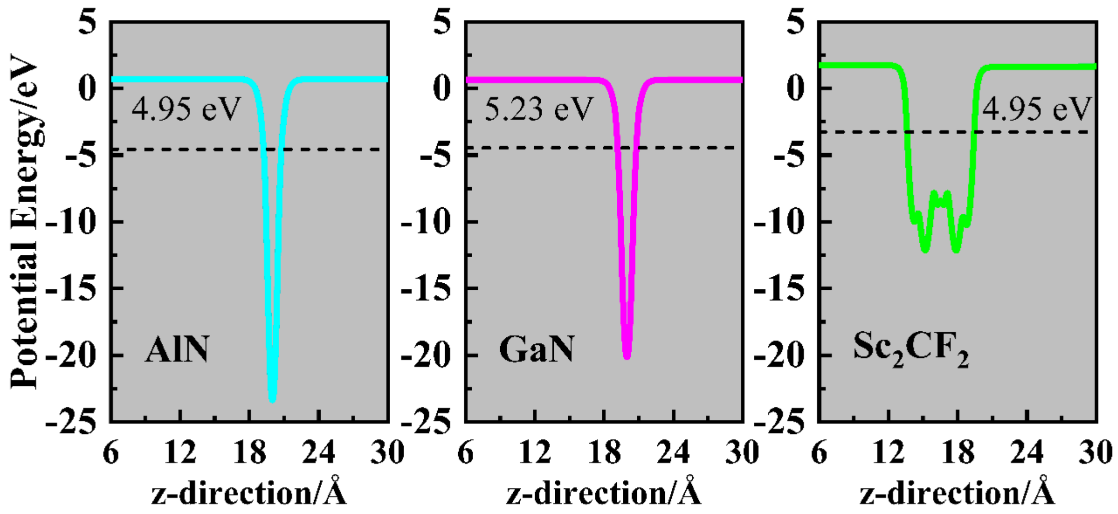


**Figure S6.** The potentials for AlN, GaN, and $Sc_2CF_2$ monolayers.

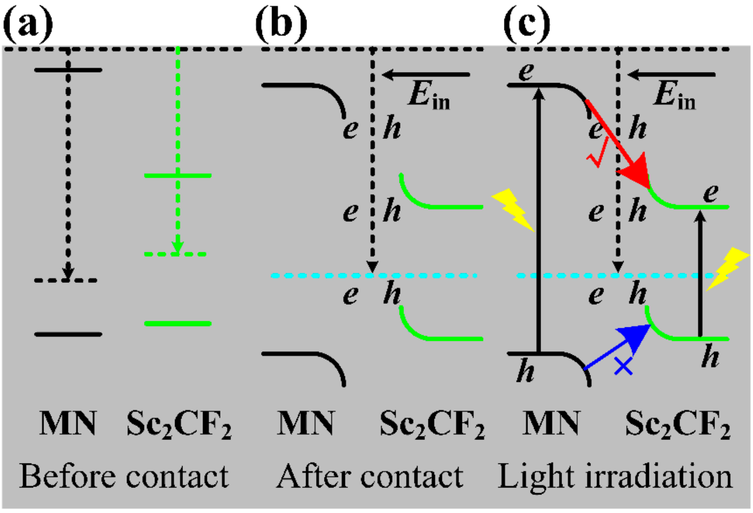


**Figure S7.** The carrier transfer mechanical of $AlN/Sc_2CF_2$ and $GaN/Sc_2CF_2$ heterostructures. The MN represents AlN and GaN.

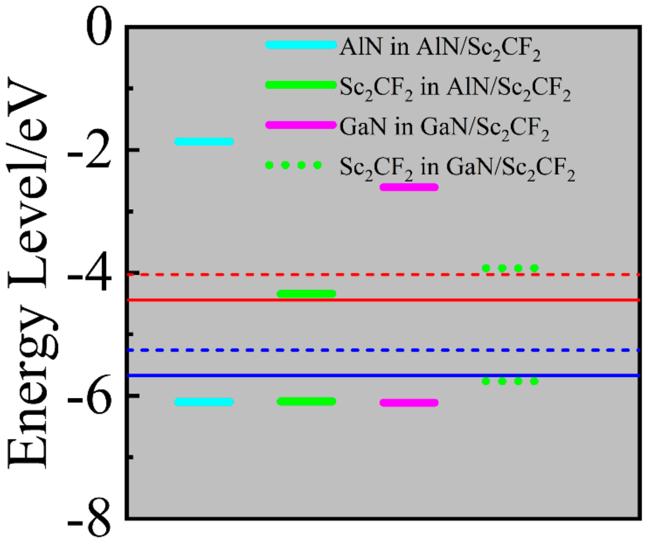


**Figure S8.** The band alignments of $AlN/Sc_2CF_2$ and $GaN/Sc_2CF_2$ heterostructures.

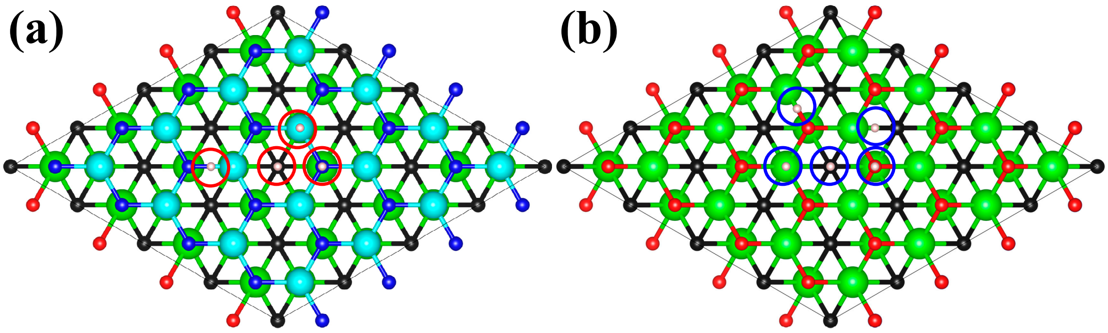


**Figure S9.** All the possible adsorption sites of H-atom on the (a) AlN layer and (b) $Sc_2CF_2$ layer in $AlN/Sc_2CF_2$ heterostructure. The same adsorption sites also have been considered in $GaN/Sc_2CF_2$ heterostructure.

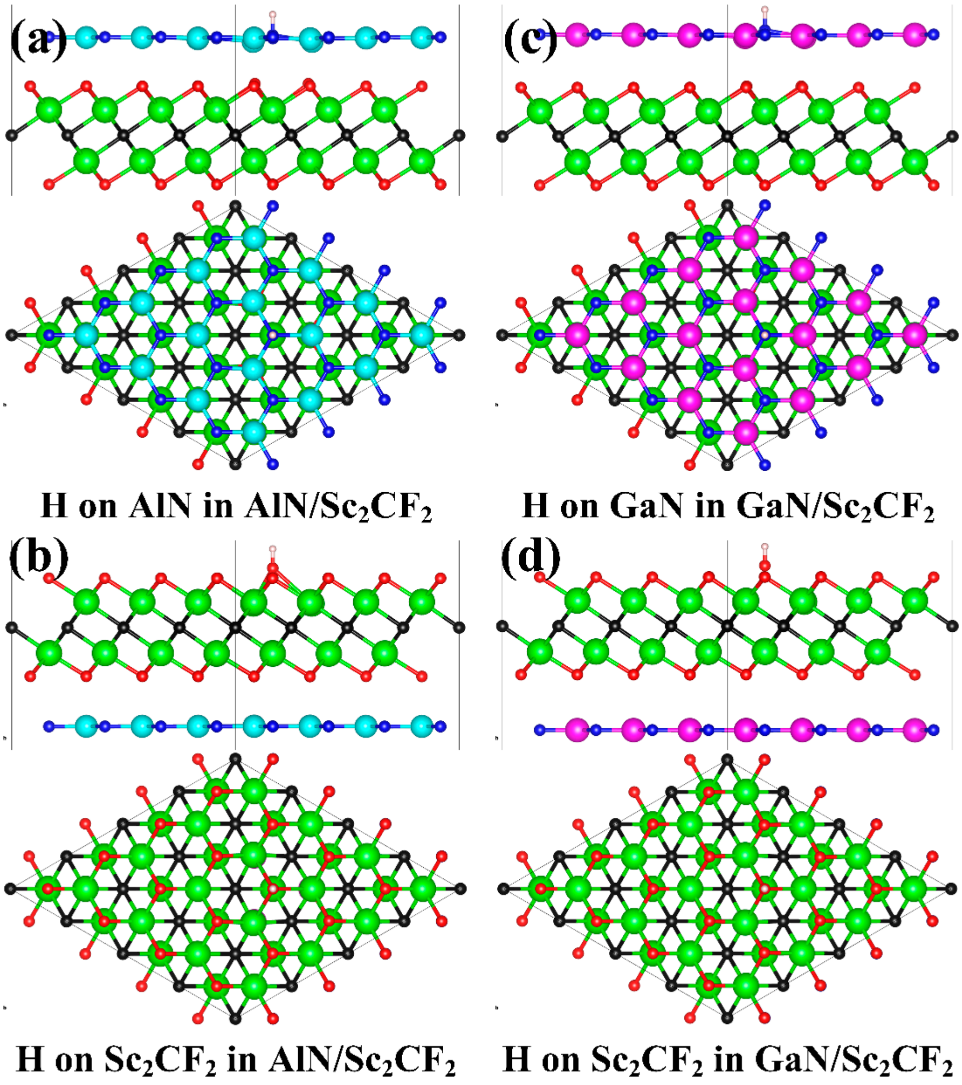


**Figure S10.** The stable adsorption of H atom on the AlN (GaN) and $Sc_2CF_2$ surface in $AlN/Sc_2CF_2$ ($GaN/Sc_2CF_2$) heterostructure.

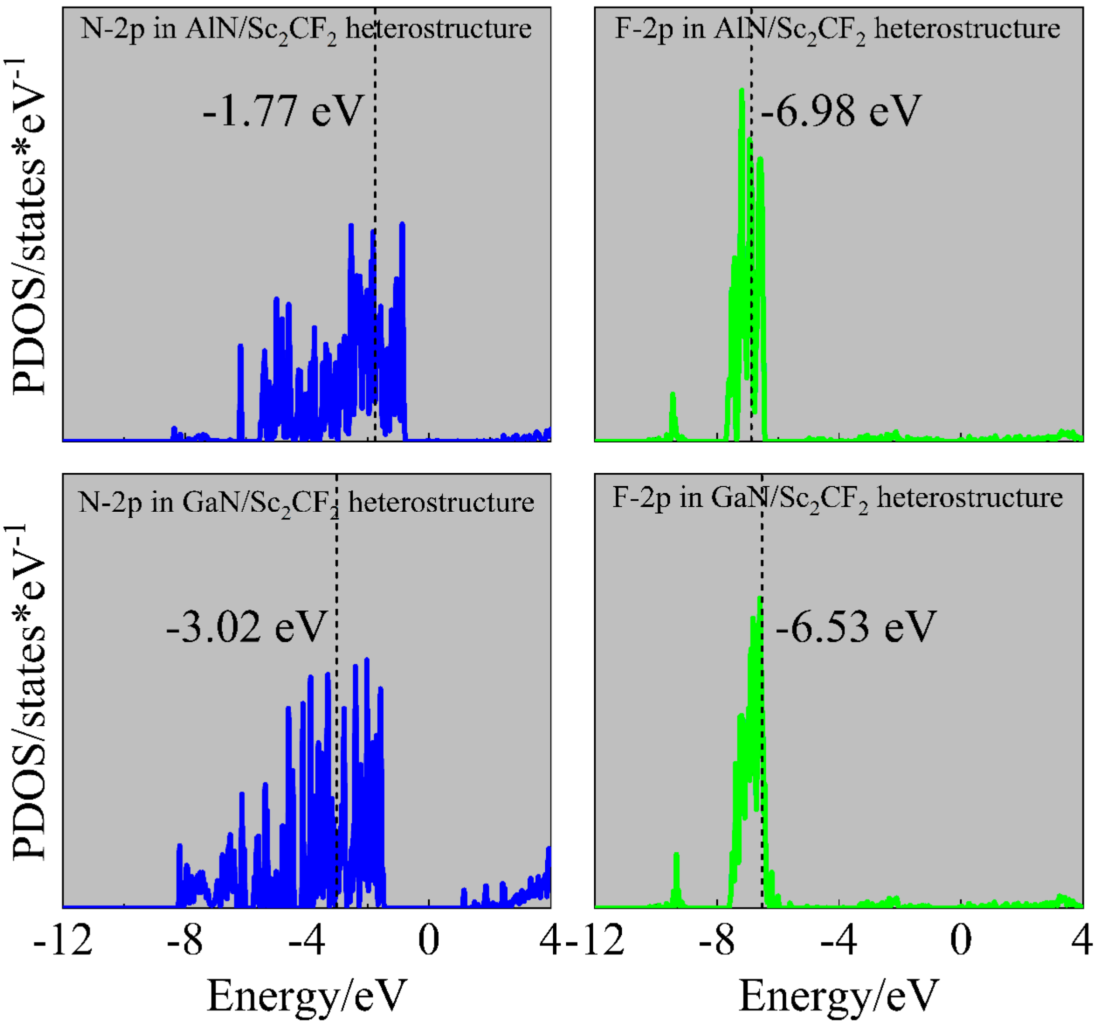


**Figure S11.** The PDOS distribution of H adsorbed on the surfaces of AlN (GaN) and $Sc_2CF_2$ in AlN/$Sc_2CF_2$ (GaN/$Sc_2CF_2$) heterostructure, respectively.

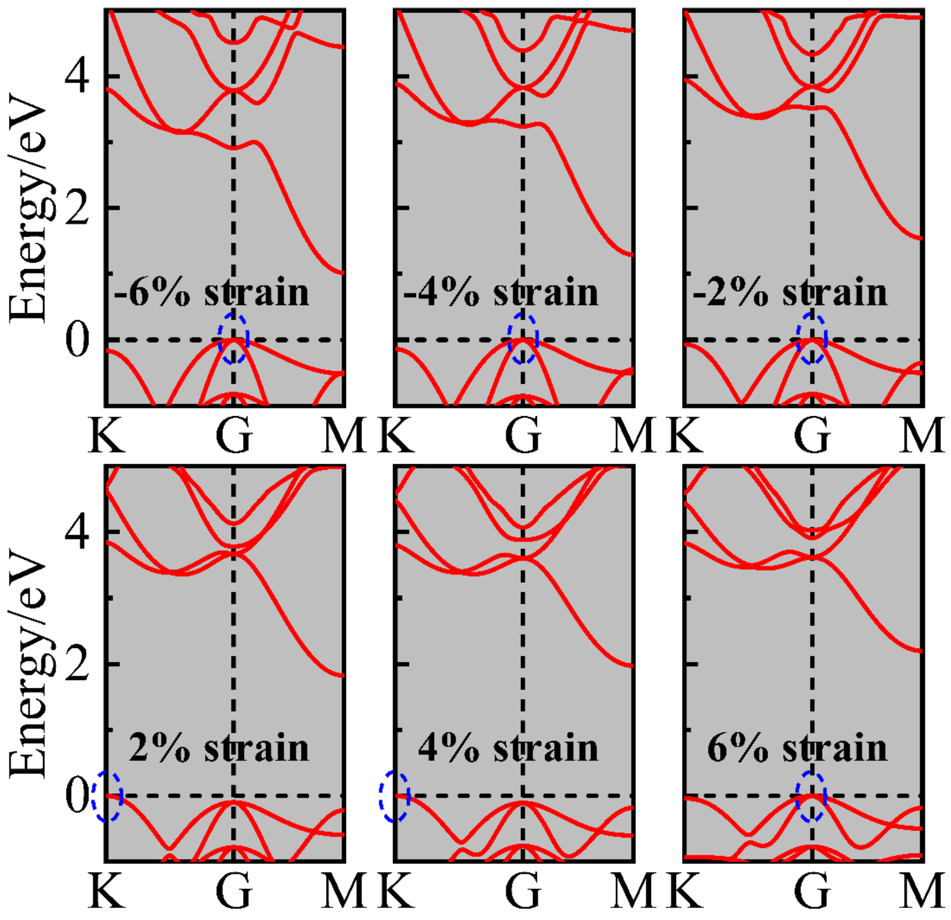


**Figure S12.** The band structures of strained $AlN/Sc_2CF_2$ heterostructures by using the HSE06 functional.

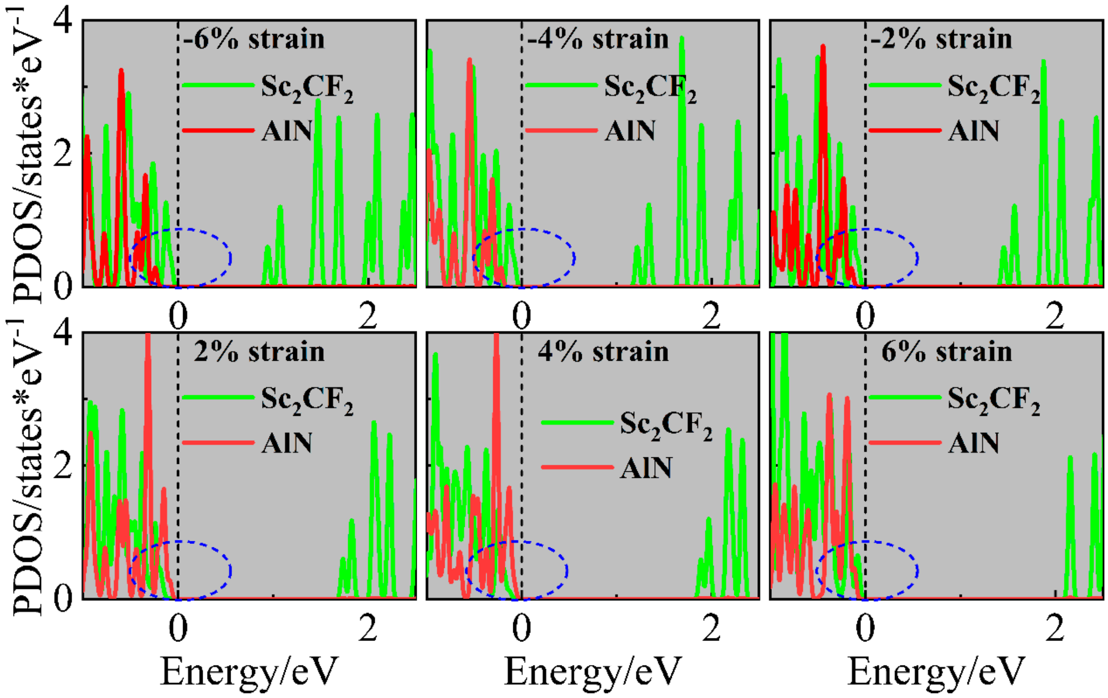


**Figure S13.** The PDOS of strained $AlN/Sc_2CF_2$ heterostructures by using the HSE06 functional.

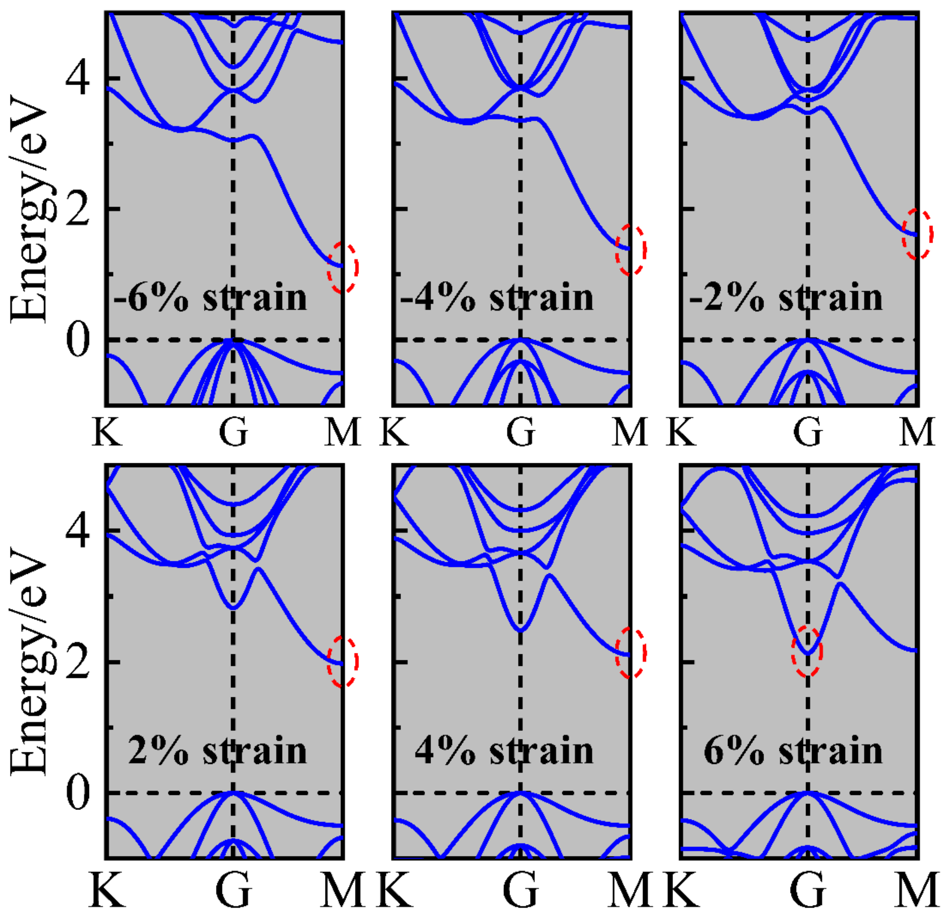


**Figure S14.** The band structures of strained GaN/$Sc_2CF_2$ heterostructures by using the HSE06 functional.

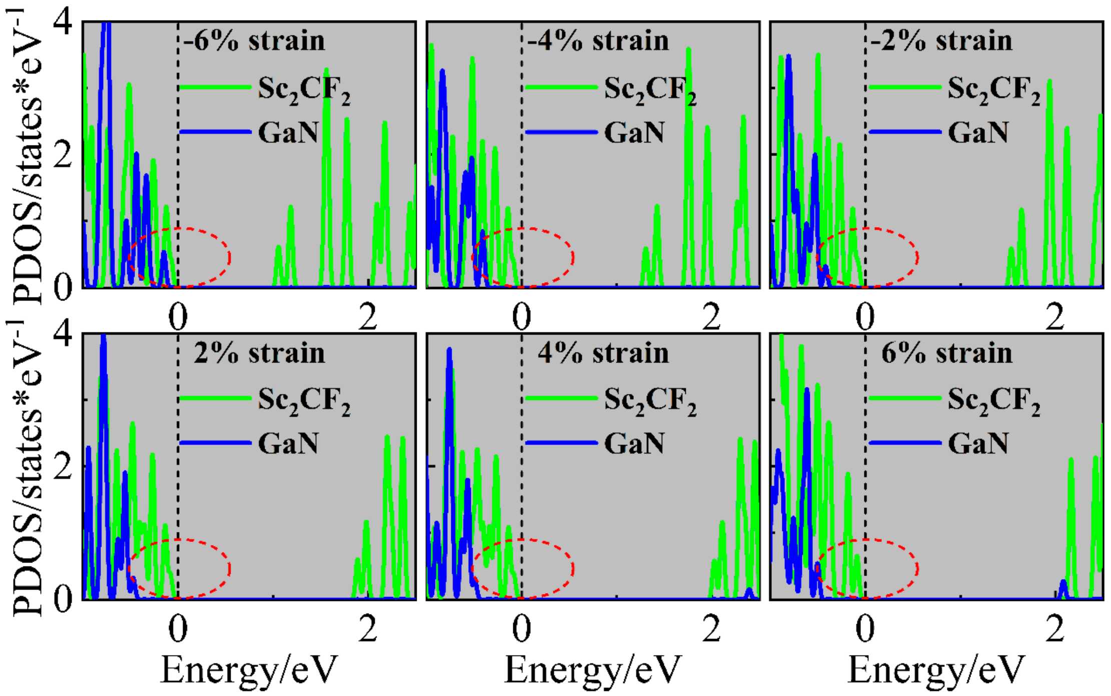


**Figure S15.** The PDOS of strained GaN/$Sc_2CF_2$ heterostructures by using the HSE06 functional.

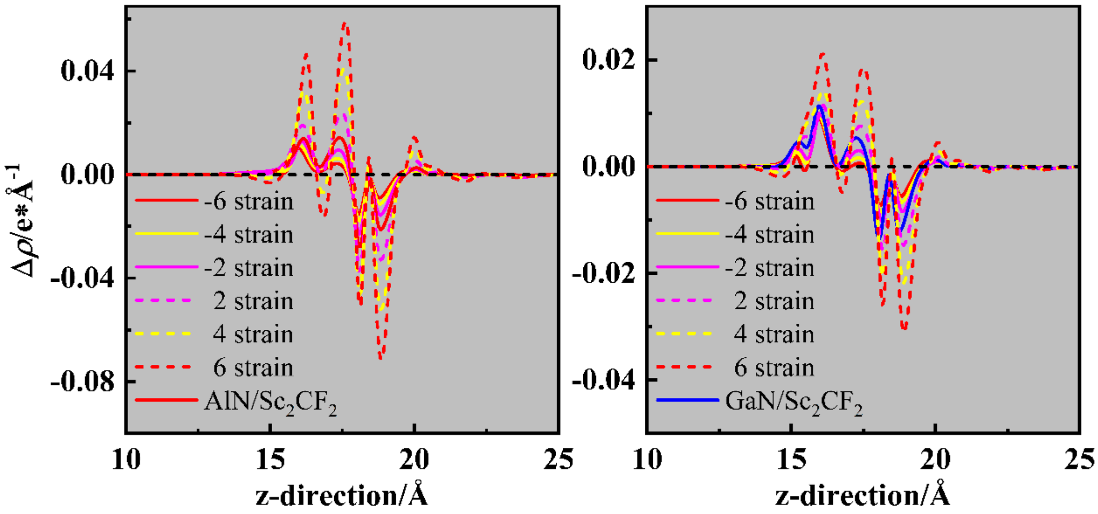


**Figure S16.** The charge density difference of the strained AlN/$Sc_2CF_2$ and GaN/$Sc_2CF_2$ heterostructures.

**Reference:**


1. Baroni, S.; De Gironcoli, S.; Dal Corso, A.; Giannozzi, P. Phonons and Related Crystal Properties from Density-Functional Perturbation Theory. *Rev. Mod. Phys.* **2001**, *73*, 515–562, doi:10.1103/RevModPhys.73.515.
2. Gonze, X.; Lee, C. Dynamical Matrices, Born Effective Charges, Dielectric Permittivity Tensors, and Interatomic Force Constants from Density-Functional Perturbation Theory. *Phys. Rev. B* **1997**, *55*, 10355–10368, doi:10.1103/PhysRevB.55.10355.
3. Togo, A.; Tanaka, I. First Principles Phonon Calculations in Materials Science. *Scripta Materialia* **2015**, *108*, 1–5, doi:10.1016/j.scriptamat.2015.07.021.
4. Ling, C.; Shi, L.; Ouyang, Y.; Zeng, X.C.; Wang, J. Nanosheet Supported Single-Metal Atom Bifunctional Catalyst for Overall Water Splitting. *Nano Lett.* **2017**, *17*, 5133–5139, doi:10.1021/acs.nanolett.7b02518.
5. Gao, Y.; Fu, C.; Hu, W.; Yang, J. Designing Direct Z-Scheme Heterojunctions Enabled by Edge-Modified Phosphorene Nanoribbons for Photocatalytic Overall Water Splitting. *J. Phys. Chem. Lett.* **2022**, *13*, 1–11, doi:10.1021/acs.jpclett.1c03527.
6. Pei, W.; Zhou, S.; Bai, Y.; Zhao, J. N-Doped Graphitic Carbon Materials Hybridized with Transition Metals (Compounds) for Hydrogen Evolution Reaction: Understanding the Synergistic Effect from Atomistic Level. *Carbon* **2018**, *133*, 260–266, doi:10.1016/j.carbon.2018.03.043.
